# Psychological Health, Polysocial Risk, and Cardiovascular Health Among Women of Reproductive Age

**DOI:** 10.1016/j.jacadv.2026.102796

**Published:** 2026-06-17

**Authors:** Faith E. Metlock, Ketum Ateh Stanislas, Dhananjay Vaidya, Melissa D. Hladek, Cheryl R. Dennison Himmelfarb, Pamela Ouyang, Jennifer Hall, Garima Sharma, Yvonne Commodore-Mensah

**Affiliations:** a Johns Hopkins School of Nursing, Baltimore, Maryland, USA; b Johns Hopkins School of Medicine, Baltimore, Maryland, USA; c American Heart Association, Dallas, Texas, USA; d Inova Heart and Vascular Institute, Fairfax, Virginia, USA; e Johns Hopkins Bloomberg School of Public Health, Baltimore, Maryland, USA

**Keywords:** cardiovascular health, psychological health, social determinants of health, women of reproductive age

## Abstract

**Background:**

Psychological health may contribute to cardiovascular health (CVH) risk among women of reproductive age, but this relationship remains understudied.

**Objectives:**

The purpose of this study was to examine the associations of depressive symptoms, anxiety symptoms, and perceived stress with adverse CVH risk factors among women of reproductive age after accounting for cumulative social disadvantage.

**Methods:**

We conducted a cross-sectional analysis of women enrolled in the SAFE HEART (Social Determinants of Hypertension Among Women of Reproductive Age) study. Depressive symptoms, anxiety symptoms, and perceived stress were assessed using the Patient Health Questionnaire-2, Generalized Anxiety Disorder-2, and Perceived Stress Scale-4, respectively. Adverse CVH risk factors were defined using Life’s Essential 8 factors. Multivariable logistic regression models adjusted for age, race/ethnicity, and polysocial risk score.

**Results:**

Among 361 women (mean age 30.87 ± 6.68 years), psychological distress was highly prevalent. Depressive symptoms, anxiety symptoms, and perceived stress were each associated with higher odds of diabetes, hypertension, hyperlipidemia, and smoking after adjustment for age, race, and cumulative social disadvantage. The strongest association was observed between depressive symptoms and smoking (adjusted OR: 4.71; 95% CI: 2.49-8.91). In contrast, low physical activity, low fruit and vegetable intake, suboptimal sleep duration, and elevated body mass index were not meaningfully associated with psychological health measures. In analyses using continuous psychological health scores, higher depressive, anxiety, and stress scores were associated with greater CVH risk, with the strongest gradient observed for depressive symptoms.

**Conclusions:**

Psychological distress was associated with several adverse CVH risk factors independent of cumulative social disadvantage, supporting more comprehensive CVH risk assessment and prevention in women of reproductive age.

Cardiovascular disease (CVD) remains the leading cause of death among women in the United States, and cardiovascular health (CVH) risk factors, such as hypertension, hyperlipidemia, and diabetes, are increasing among women of reproductive age.^1^, ^2^, ^3^, ^4^ These trends are concerning because suboptimal CVH in early and mid-adulthood is associated with both future CVD and adverse pregnancy outcomes, including preeclampsia, gestational hypertension, and preterm birth.^5^^,^^6^ Despite public health efforts to reduce CVD-related morbidity and mortality among women of reproductive age, these disparities persist, particularly among racially and socioeconomically diverse populations,^7^^,^^8^ underscoring the need for a more comprehensive understanding of additional contributing factors.

Social determinants of health are important drivers of cardiovascular disparities, but they may not fully explain the disproportionate burden of CVH risks among women of reproductive age.^3^^,^^9^^,^^10^ This gap highlights the importance of examining additional mechanisms, such as psychological health, which has received comparatively less attention in cardiovascular research. Depression, anxiety, and stress have been linked to adverse cardiovascular outcomes through physiologic mechanisms, including autonomic dysfunction, inflammation, and hypothalamic-pituitary-adrenal axis dysregulation, as well as through health behaviors such as smoking, poor diet, and physical inactivity.^11^, ^12^, ^13^, ^14^, ^15^, ^16^ However, few studies have examined these relationships among women of reproductive age in community-based samples, where social disadvantage and psychological distress may coexist and jointly shape CVH risk.^17^

The present study examines the associations between psychological health, including depression symptoms, anxiety symptoms, and perceived stress, and CVH risk, as defined by Life’s Essential 8^18^, among a diverse sample of women aged 18 to 50, recruited through community-based approaches in Maryland and Washington, DC. To account for the broader social context in which psychological distress occurs, we additionally adjusted for polysocial risk scores, a composite measure of cumulative social disadvantage capturing multiple domains of social risk.^19^ We sought to determine whether associations between psychological health and CVH risk factors persist independent of cumulative social risk.

## Methods

**Figure 1:**
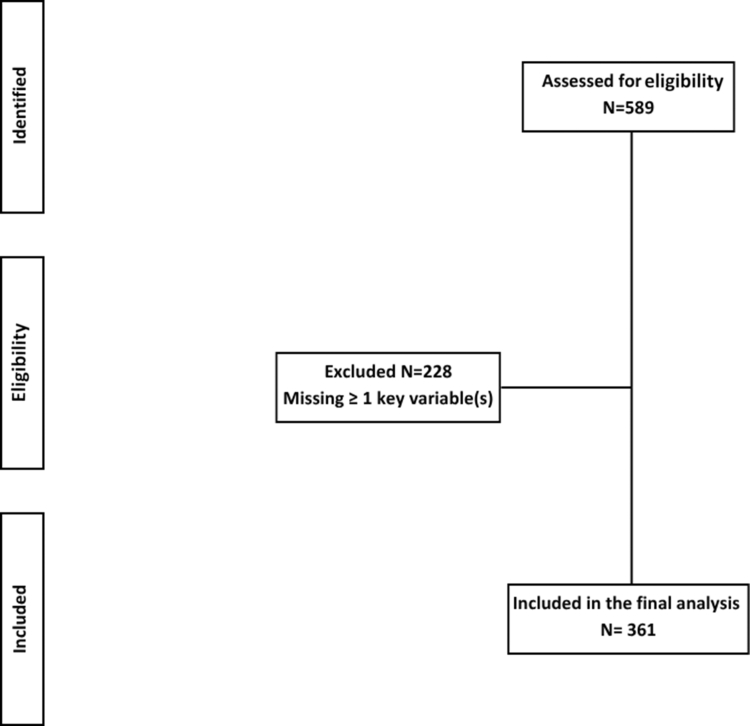
**Study Flowchart** Flowchart depicting participant recruitment and data inclusion for the study. A total of 589 women of reproductive age were assessed for eligibility from community-based settings in the Baltimore and Washington, DC area. After excluding 228 participants due to missing key variables, the final analytical sample consisted of 361 women with complete data.

The methods for the SAFE HEART (Social Determinants of Hypertension Among Women of Reproductive Age) study have been described in detail elsewhere.^20^ Briefly, this study examined the associations between psychological health and CVH risk factors among women of reproductive age. Over a one and a half-year study period (April 2023–November 2024), we recruited 589 women from the Baltimore and Washington, DC, metropolitan area through community-based outreach efforts. Eligible participants were between 18 and 50 years old, self-identified as women, and were able to speak and read English or Spanish. We excluded participants with missing key variables (n = 228), yielding a final analytical sample of 361 (Figure 1).

### Psychological health

Psychological health was measured using 3 validated self-report instruments: the Generalized Anxiety Disorder-2^21^ (GAD-2), the Patient Health Questionnaire-2^22^ (PHQ-2), and the Perceived Stress Scale-4^23^ (PSS-4). The GAD-2 and PHQ-2 each yield scores ranging from 0 to 6, with scores ≥3 indicating elevated anxiety and depressive symptoms, respectively.^21^^,^^22^ The PSS-4 yields scores ranging from 0 to 16, with higher scores reflecting greater perceived stress.^23^ Given the absence of a validated diagnostic cutoff for the PSS-4, perceived stress was analyzed as a continuous measure in regression models and dichotomized at the sample median (≥8) for descriptive analyses only. Additional instrument details are provided in Supplemental Table 1.

### Cardiovascular health risk factors

CVH risk, as defined by Life’s Essential 8,^18^ was assessed using self-reported CVH risk factors. Participants were asked whether they had ever been diagnosed with hypertension, diabetes, or hyperlipidemia by a physician. Smoking status was determined by asking whether participants had smoked at least 100 cigarettes in their lifetime. Physical activity was classified based on self-reported weekly exercise, with <150 minutes of moderate-intensity activity per week considered adverse. Dietary intake was assessed based on daily fruit and vegetable consumption, with <5 servings per day classified as suboptimal. Sleep duration was categorized as adverse if participants reported sleeping <7 hours or >9 hours per night. Body mass index (BMI) was calculated using self-reported height and weight, with a BMI ≥25 kg/m^2^ considered an adverse cardiovascular risk factor. The total number of adverse CVH risk factors was summed for each participant, ranging from 0 to 8.

### Polysocial risk

Polysocial risk was measured using a composite polysocial risk score, which captured 14 distinct social risk factors across 6 domains: socioeconomic stability (education, employment, income, insurance, and financial strain), living situation (housing stability, housing quality, marital status, and homeownership), food security, transportation, utilities, and interpersonal safety using the Accountable Health Communities Screening Tool.^24^ Each unfavorable social condition (eg, financial strain, housing instability) was assigned one point, while favorable conditions were assigned zero points. The final polysocial risk score ranged from 0 to 14, with higher scores indicating a greater burden of social risk factors (Supplemental Table 2).^9^

### Statistical analysis

Descriptive analyses summarized the prevalence of psychological health conditions and CVH risk factors in the study population. Associations between psychological health measures and individual adverse CVH risk factors were evaluated using multivariable logistic regression. Depressive symptoms and anxiety symptoms were modeled dichotomously using validated PHQ-2 and GAD-2 cutoffs (scores ≥3), whereas perceived stress was modeled continuously using the PSS-4 because no validated clinical threshold exists. Adjusted models included age category (18-25, 26-34, and 35-50 years), self-reported race, and polysocial risk score, selected a priori using a directed acyclic graph (Supplemental Figure 1). Age was categorized to address nonlinearity identified in preliminary model diagnostics. All primary models met prespecified diagnostic criteria, and sensitivity analyses addressing residual nonlinearity in PSS-4 did not materially change study conclusions (Supplemental Table 3). To evaluate cumulative CVH burden, we examined linear associations between continuous psychological health scores (PHQ-2, GAD-2, and PSS-4) and the total number of adverse CVH risk factors. Heat maps were generated to visualize the joint distribution of psychological health scores, polysocial risk score, and number of CVH risk factors. Results are presented as ORs with 95% CIs. All analyses were performed using Stata version 18.0 (StataCorp) and R version 4.4.1 (R Foundation for Statistical Computing). A 2-sided *P* value <0.05 was considered statistically significant.

### Ethical approval

This study was approved by the Johns Hopkins Medicine Institutional Review Board under IRB number IRB00337704.

## Results

### Sociodemographic characteristics

The analytic sample included 361 women with a mean age of approximately 31 years, of whom a small proportion were pregnant or lactating. Participants represented diverse racial and ethnic backgrounds, with nearly half identifying as non-Hispanic Black, followed by Hispanic/other, non-Hispanic White, non-Hispanic Asian, and American Indian/Alaskan Native. Over half were single, and the remainder were married or cohabitating. Educational attainment was predominantly high school level or above, and household income varied, with representation across both lower- and higher-income groups. Financial strain was frequently reported. Most participants were insured and employed, though a smaller portion reported unemployment. Housing instability and housing-related challenges were common. Food insecurity, concerns about running out of food, and limited means to purchase more were frequently endorsed. Additionally, transportation barriers, utility threats, and concerns about safety in interpersonal environments were reported by a notable share of participants (Table 1, Supplemental Table 4).

**Table 1:** Sociodemographic and Social Risks (N = 361)

Age, y	30.87 ± 6.68
18-25	80 (22.16%)
26-34	191 (52.91%)
35-50	90 (24.93%)
Pregnant/lactating	38 (10.53%)
Race/ethnicity	
Non-Hispanic White	83 (22.99%)
Non-Hispanic Black	170 (47.09%)
Non-Hispanic Asian	12 (3.32%)
American Indian-Alaskan Native	2 (0.55%)
Hispanic/other	94 (26.04%)
Marital status	
Single	191 (52.91%)
Education	
≤ High school graduate	73 (20.22%)
Household income	
≤ $50,000	156 (43.21%)
Financial strain	
Hard to pay for food, housing, medical care, and heating	222 (61.50%)
Insurance	
Uninsured	74 (20.50%)
Employment	
Unemployed	47 (13.02%)
Living situation	
Rent/other arrangement	247 (68.42%)
Unsteady place	79 (21.88%)
Housing problems	234 (64.82%)
Food insecurity	
Worried food would run out	189 (52.35%)
No money to get more food	183 (50.69%)
Transportation	
Lack of reliable transportation	117 (32.41%)
Utilities	
Threatened to shut off services	90 (24.93%)
Interpersonal safety	
Unsafe	72 (19.94%)
Polysocial risk score	6.00 ± 2.91

### Psychological and cardiovascular health risk factors

Participants reported varying levels of psychological distress, with notable proportions screening positive for depressive symptoms, anxiety symptoms, and perceived stress. Cardiometabolic conditions, including hypertension, hyperlipidemia, and diabetes, were frequently reported. Smoking history was present among a substantial subset of participants. Lifestyle-related behaviors such as low physical activity, limited fruit and vegetable intake, elevated body weight, and suboptimal sleep duration were also common (Table 2, Supplemental Figure 2).

**Table 2:** Health Risk Factors (N = 361)

Psychological health	
Depressive symptoms^a^	
Dichotomized (≥3 high)	105 (29.09%)
Continuous (0-6)	1.70 ± (1.55)
Anxiety symptoms^b^	
Dichotomized (≥3 high)	129 (35.73%)
Continuous (0-6)	1.96 ± 1.70
Stress^c^	
Dichotomized (≥8 high)	229 (63.43%)
Continuous (0-16)	6.63 ± 3.21
Cardiovascular health^d^	
Diabetes	104 (28.81%)
Hyperlipidemia	106 (29.36%)
Hypertension	127 (35.18%)
Smoked 100 cigarettes in lifetime	88 (24.38%)
Low physical activity (<150 min/moderate)	285 (78.95%)
Low fruit and vegetable intake	276 (76.45%)
High body mass index (≥25 kg/m^2^)	279 (77.29%)
<7 h and > 9 h of sleep	181 (50.14%)
Cardiovascular health risk factors	2.45 ± 1.54

GAD-2 = Generalized Anxiety Disorder-2; PHQ-2 = Patient Health Questionnaire-2; PSS-4 = Perceived Stress Scale-4.

a PHQ-2 score.

b GAD-2 score.

c PSS-4 score.

d Defined by Life’s Essential 8.

### Psychological health by cardiovascular risk factors

**Figure 2:**
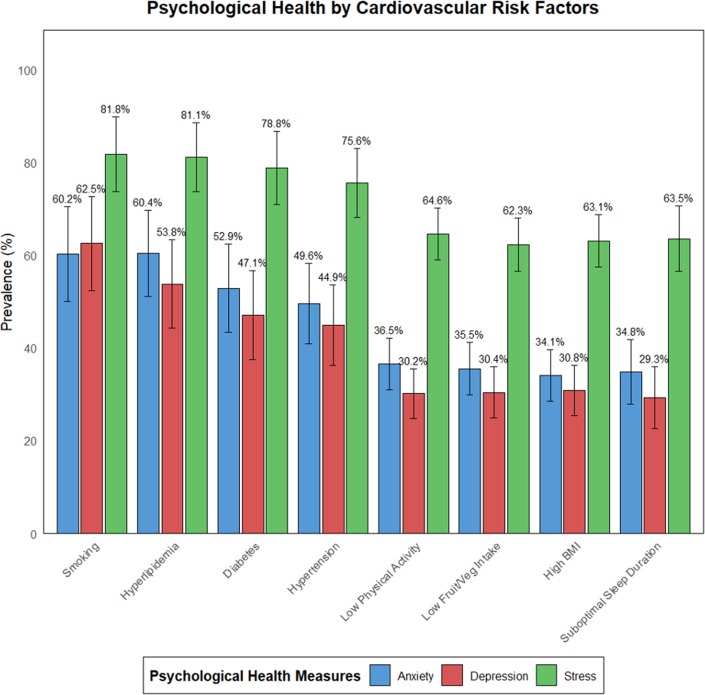
**Distribution of Psychological Health by Cardiovascular Risk Factors** Prevalence of high depressive symptoms, high anxiety symptoms, and high perceived stress across cardiovascular health risk factors as defined by Life’s Essential 8. Psychological health measures were dichotomized: anxiety (Generalized Anxiety Disorder-2 score ≥3), depression (Patient Health Questionnaire-2 score ≥3), and stress (Perceived Stress Scale-4 score ≥8), corresponding to the sample median. BMI = body mass index.

Women with underlying cardiometabolic conditions reported higher levels of psychological distress across all domains. Those with hyperlipidemia, diabetes, and hypertension consistently showed elevated prevalence of perceived stress, depressive symptoms, and anxiety symptoms. Smoking history was also associated with higher psychological distress across all 3 outcomes, while elevated BMI demonstrated comparatively lower levels of distress relative to other health conditions. Across lifestyle behaviors such as sleep, diet, and physical activity, variations in depressive symptoms and anxiety symptoms were modest, although levels of perceived stress remained high (Figure 2).

### Association between psychological health and Life’s Essential 8

**Figure 3:**
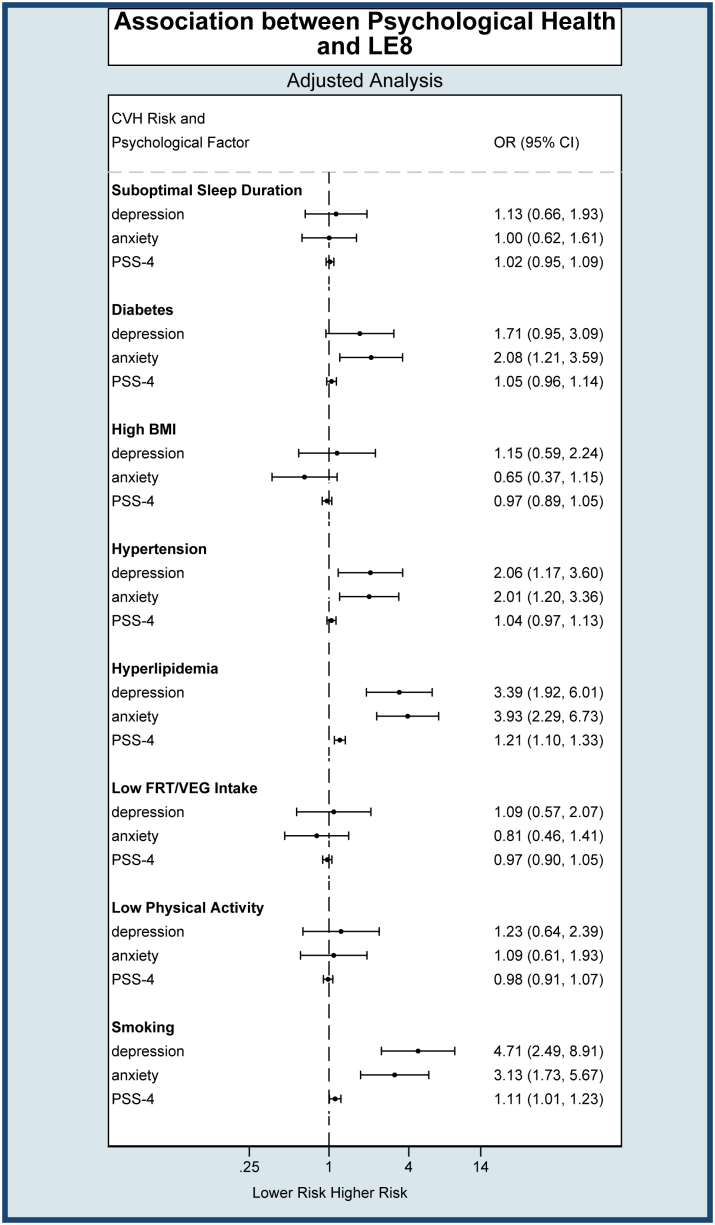
**Association Between Psychological Health and Life’s Essential 8 Cardiovascular Risk Factors** Adjusted associations between psychological health measures (high depressive symptoms, high anxiety symptoms, and high perceived stress) and cardiovascular health risk factors as defined by Life’s Essential 8. Models were adjusted for polysocial risk scores (range: 0-14) to account for cumulative social disadvantage. Cardiovascular health risk factors were dichotomized as follows: hypertension, diabetes, and hyperlipidemia (self-reported physician diagnosis), smoking (≥100 cigarettes lifetime), physical activity (<150 minutes of moderate-intensity exercise per week), diet (<5 servings of fruits and vegetables per day), body mass index (≥25 kg/m^2^), and sleep (<7 or >9 hours per night). Psychological health measures were modeled as follows: anxiety symptoms (Generalized Anxiety Disorder-2 score ≥3) and depressive symptoms (Patient Health Questionnaire-2 score ≥3) were dichotomized, whereas perceived stress was modeled continuously using the Perceived Stress Scale-4. Odds ratios for perceived stress represent the change in odds associated with each one-point increase in the PSS-4 score. CVH = cardiovascular health; PSS-4 = Perceived Stress Scale-4; other abbreviation as in Figure 2.

Women reporting high depressive symptoms showed varying relationships with Life’s Essential 8 metrics. Behavioral factors such as sleep duration, fruit and vegetable intake, and physical activity displayed little variation between those with and without high depressive symptoms. In contrast, smoking emerged as a notable behavioral correlate, demonstrating one of the strongest relationships with depressive symptoms. Cardiometabolic conditions, including diabetes, hypertension, and hyperlipidemia, were consistently elevated among women with depressive symptoms. High BMI, however, did not show a meaningful pattern in relation to depressive symptoms. Among women experiencing high anxiety symptoms, lifestyle behaviors such as sleep, diet, and physical activity showed minimal variability. Smoking, however, remained a marked behavioral correlate, with anxiety symptomatic women displaying higher likelihood of smoking behaviors compared to those without anxiety symptoms. Cardiometabolic health factors presented a clear pattern: diabetes, hypertension, and hyperlipidemia were more prevalent among women with high anxiety symptoms. As with high depressive symptoms, elevated BMI did not correspond to higher anxiety symptoms. Perceived stress followed trends similar to depression and anxiety, with limited differentiation by sleep patterns, diet quality, or physical activity. These behaviors did not demonstrate consistent directional relationships. Smoking retained its position as a key behavioral marker, remaining more common among women with heightened stress. Cardiometabolic conditions, particularly diabetes, hypertension, and hyperlipidemia, were again elevated among women experiencing stress. BMI did not exhibit a clear association with perceived stress. These trends were consistent across crude estimates presented in Supplemental Figure 3 and remained evident in the adjusted models (Figure 3, Supplemental Tables 5 and 6).

### Association between continuous mental health scores and cardiovascular risk factors

**Figure 4:**
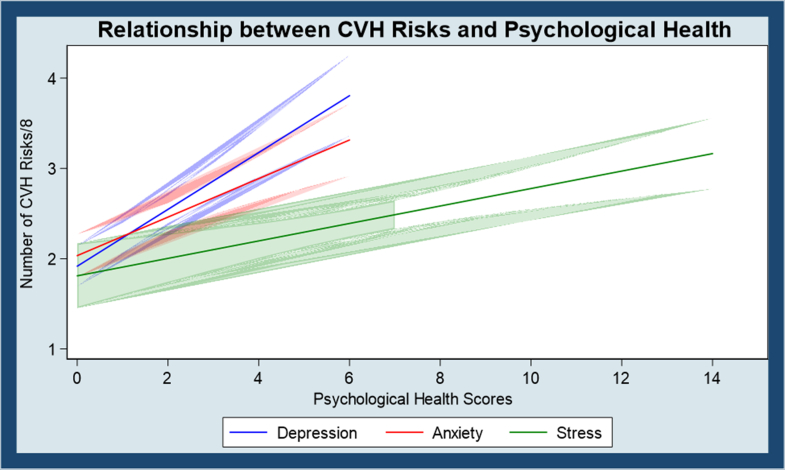
**Cardiovascular Risk Burden by Psychological Health Scores** The area line graph displays the relationship between psychological health scores (x-axis, ranging from 0 to 14) and the number of cardiovascular health risks (y-axis, ranging from 0-8) for 3 different measures: depression (Patient Health Questionnaire-2, blue: 0-6), anxiety (Generalized Anxiety Disorder-2, red: 0-6), and perceived stress (Perceived Stress Scale-4, green: 0-16). The solid lines represent predicted values from linear regression models, while the shaded areas around each line represent 95% CIs for these predictions, with depression and anxiety scores ranging from 0 to 6 and stress scores ranging from 0-16 for study participants. Cardiovascular risk factors were assessed using Life’s Essential 8, including hypertension, diabetes, hyperlipidemia, smoking, low physical activity (<150 min/wk), suboptimal diet (<5 servings of fruits/vegetables per day), suboptimal sleep duration (<7 or >9 h/night), and overweight/obesity (body mass index ≥25 kg/m^2^) Abbreviation as in Figure 3.

The association between continuous mental health scores and the number of CVH risk factors revealed distinct patterns across depressive symptoms, anxiety symptoms, and stress (Figure 4, Supplemental Figure 4). Depressive symptoms exhibited the steepest increase in CVH risk factors as scores increased, followed by anxiety symptoms, while stress showed a more gradual increase. Heat maps illustrate the relationship between polysocial risk, mental health scores, and CVH risk factors. CVH risk factors were prevalent across all levels of polysocial risk but increased substantially at higher scores. For stress (Supplemental Figure 5), CVH risk factors were most pronounced when polysocial risk scores exceeded 5, with the highest burden occurring at scores greater than 7. For depression (Supplemental Figure 6), cardiovascular risk factors were evident even at low polysocial risk scores (≥2) but peaked when depressive symptom scores exceeded 3 and polysocial risk exceeded 6. Similarly, anxiety (Supplemental Figure 7) was associated with increased CVH risk at all levels of polysocial risk, with the highest burden appearing when anxiety symptom scores exceeded 2 and polysocial risk was >4 (Central Illustration).

**Central Illustration:**
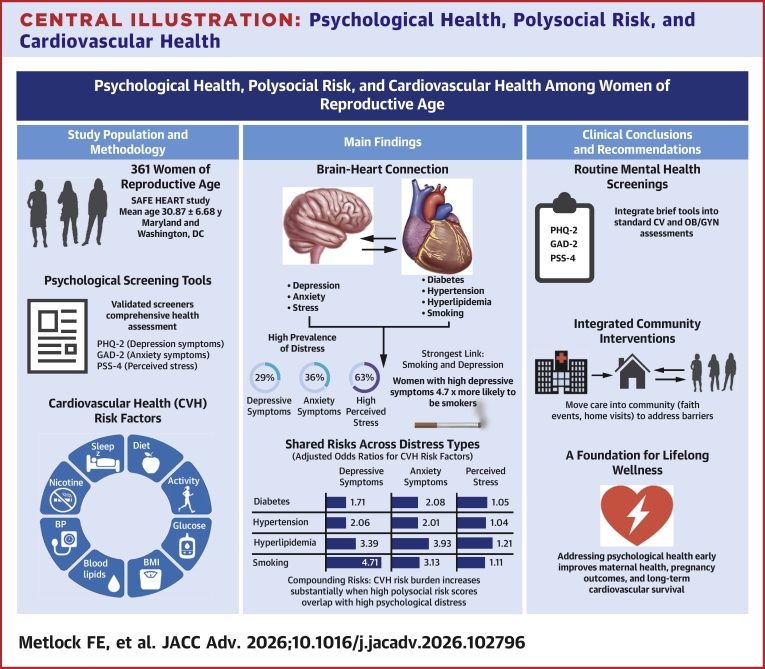
**Psychological Health, Polysocial Risk, and Cardiovascular Health** This illustration summarizes the SAFE HEART (Social Determinants of Hypertension Among Women of Reproductive Age) study of 361 women of reproductive age from Maryland and Washington, DC. Participants completed validated assessments of depressive symptoms (Patient Health Questionnaire-2), anxiety symptoms (Generalized Anxiety Disorder-2), and perceived stress (Perceived Stress Scale-4), along with measures of adverse cardiovascular health risk factors. Psychological distress was highly prevalent and was associated with greater odds of diabetes, hypertension, hyperlipidemia, and smoking, with the strongest association observed between depressive symptoms and smoking. The figure also highlights the compounding burden of high psychological distress and high polysocial risk on cardiovascular health risk. These findings support routine psychological health screening and integrated community-based strategies to improve cardiovascular health prevention in women of reproductive age. GAD-2 = Generalized Anxiety Disorder-2; PHQ-2 = Patient Health Questionnaire-2; other abbreviations as in Figures 2 and 3.

## Discussion

Among women of reproductive age in the Maryland and Washington, DC area, psychological distress, including depressive symptoms, anxiety symptoms, and perceived stress, was highly prevalent and associated with several adverse CVH risk factors. Notably, these associations persisted even after accounting for cumulative social disadvantage, underscoring that psychological health is an independent yet deeply intertwined contributor to cardiovascular disparities. Heat map analyses further revealed that although CVH risk factors were present across all levels of cumulative social disadvantage, CVH burden increased as psychological health scores worsened. Taken together, these findings support the inclusion of psychological health in more comprehensive approaches to CVH risk assessment and prevention among women of reproductive age.

Depression among women of reproductive age is a significant public health concern and has been associated with early cardiometabolic risk, including hypertension, hyperlipidemia, and diabetes.^14^^,^^25^ A nationally representative study further underscore this concern, showing a high burden of depression among women of reproductive age, with hypertension emerging as a key cardiovascular risk factor among those with depressive symptoms.^26^ Our study aligns with these findings, demonstrating significant associations between depression risk and multiple CVH risk factors, even after adjusting for cumulative social disadvantage. This reflects a complex interplay between psychological health, environmental stressors, and CVH risk, wherein social disadvantage and psychological distress contribute to unhealthy behaviors such as smoking, which then accelerate the development of CVH risk factors.^27^ These findings support the value of incorporating brief depression screening, such as the PHQ-2, into CVH assessment to identify women who may benefit from earlier risk reduction efforts.^22^

Perceived stress was the most prevalent form of psychological distress in our sample and was associated with diabetes, hypertension, hyperlipidemia, and smoking, independent of cumulative social disadvantage. These findings align with prior work showing that social disadvantage and psychosocial stress contribute to racial and ethnic differences in CVH and may be especially consequential for women.^3^^,^^10^^,^^28^ Sex-specific hormonal and autonomic responses to stress may further exacerbate CVH risk, making stress a critical yet often overlooked factor in women’s CVH.^16^^,^^29^^,^^30^ In view of the widespread burden of stress among women of reproductive age and its strong links to CVH risk factors, incorporating psychological health screening into routine CVH assessments and developing innovative stress reduction interventions is essential for improving long-term cardiovascular outcomes in this population.

Anxiety symptoms were also associated with adverse CVH risk in this study, even after accounting for cumulative social disadvantage. Prior research suggests that anxiety may contribute to CVH risks through activation of the hypothalamic-pituitary-adrenal axis, sympathetic nervous system dysregulation, and endothelial dysfunction.^14^ Anxiety may be particularly relevant for women’s CVH, given evidence linking pregnancy-related anxiety with adverse pregnancy outcomes and long-term CVD.^31^ Since women of reproductive age face unique social and biological stressors, it is critical to recognize the role of anxiety as both a physiological and behavioral mechanism that can further exacerbate CVH risk. Future efforts should prioritize normalizing anxiety while equipping women with the tools to navigate stressors effectively, ultimately mitigating their long-term CVH risk.^32^^,^^33^

Clinical and public health efforts must move beyond traditional CVD risk assessments to incorporate routine mental health screening, such as the PHQ-2, GAD-2, and PSS-4, and targeted interventions that address both psychological distress and CVH risk factors. Primary care clinicians, including obstetricians and gynecologists, may be particularly well positioned to incorporate these assessments into preventive care. However, implementation may be limited by mental health stigma, workforce shortages,^34^ long wait times, and insufficient referral infrastructure.^35^^,^^36^ Expanding community-based mental health resources, such as stress management workshops, peer support groups, and culturally tailored interventions,^37^ could help bridge gaps in access to care and reduce disparities in both psychological and CVH.^38^ Leveraging mobile-based mental health interventions may also provide an innovative solution for reaching women in diverse settings. Future research should explore the longitudinal impact of depression, anxiety, and stress on CVD risk, as well as assess the effectiveness of various interventions in mitigating these risks. Additionally, research must account for the intersectionality of psychological health with reproductive milestones, including pregnancy, postpartum recovery, and menopause, to better understand how mental health fluctuations over the life course impact CVH risk trajectories. Continued inclusion of racially, ethnically, and socioeconomically diverse populations will be essential for understanding how social and psychological factors jointly shape CVH risk in women.

### Study Limitations

While this study provides valuable insights into the relationship between psychological health and CVH risks among women of reproductive age, several limitations should be acknowledged. First, its cross-sectional design prevents causal inference, making it unclear whether psychological distress contributes to CVH risk or vice versa. Future longitudinal studies are needed to clarify these relationships. Second, self-reported measures for psychological health and CVH risk factors may be subject to recall and social desirability bias. In particular, diabetes, hypertension, and hyperlipidemia were based on self-reported prior diagnosis, which may have underestimated disease burden by excluding undiagnosed cases that would require objective clinical or laboratory testing to detect. Although validated screening tools (PHQ-2, GAD-2, and PSS-4) were used, they do not provide clinical diagnoses. Objective assessments, such as blood pressure, lipid panels, and glucose tests, would strengthen future studies. Third, while we adjusted for cumulative social disadvantage, unmeasured social and environmental factors (eg, workplace stress, neighborhood conditions, and health care access) may still confound findings. Future studies should incorporate broader social determinants of health to provide a more comprehensive understanding. Despite these limitations, our findings emphasize the urgent need for early screening and targeted interventions to address cardiovascular disparities in this population.

## Conclusions

This study highlights the associations between psychological distress and CVH risk among women of reproductive age, emphasizing the need for integrated approaches that address both mental and CVH. By incorporating psychological health screening, expanding community-based interventions, and prioritizing research on effective prevention strategies, we can work toward reducing cardiovascular disparities and improving long-term health outcomes for women. Addressing psychological health as part of comprehensive cardiovascular care has the potential to significantly impact the lives of younger women, setting the foundation for better maternal health, pregnancy outcomes, and lifelong cardiovascular wellness.

Perspectives **COMPETENCY IN MEDICAL KNOWLEDGE:** Psychological distress, including depressive symptoms, anxiety symptoms, and perceived stress, was associated with several adverse cardiovascular health risk factors among women of reproductive age, even after accounting for cumulative social disadvantage. Brief psychological health screening may strengthen cardiovascular risk assessment in this population.

**TRANSLATIONAL OUTLOOK:** Future studies should evaluate integrated clinical and community-based strategies that combine psychological health screening with cardiovascular prevention and referral pathways. Longitudinal research is also needed to clarify how psychological distress and social risks jointly influence cardiovascular health across reproductive life stages.

## Funding support and author disclosures

This study was funded by the 10.13039/100000968American Heart Association's Research Goes Red Grants 979462 and 147554. Dr Metlock is supported by the 10.13039/100000056National Institute of Nursing Research of the 10.13039/100000002National Institutes of Health under award Number T32NR020315 and the 10.13039/100000968American Heart Association under award number 24PRE12438460. Dr Commodore-Mensah is supported by the 10.13039/100000968American Heart Association Health Equity Research Network on the Prevention of Hypertension (Grant number: 882415) and 10.13039/100006545National Institute on Minority Health and Health Disparities (Grant number: P50MD017348-818). Dr Sharma is supported by the 10.13039/100000968American Heart Association under award number 979462. The authors have reported that they have no relationships relevant to the contents of this paper to disclose.
