# Supplementary material for: Psychological Health, Polysocial Risk, and Cardiovascular Health Among Women of Reproductive Age
Source: JACC Adv. 2026 Jun 17;5(6):102796. doi: 10.1016/j.jacadv.2026.102796 (PMC13309340; doi:10.1016/j.jacadv.2026.102796)
Supplement: Supplementary material [file mmc1.pdf]

# Supplemental File

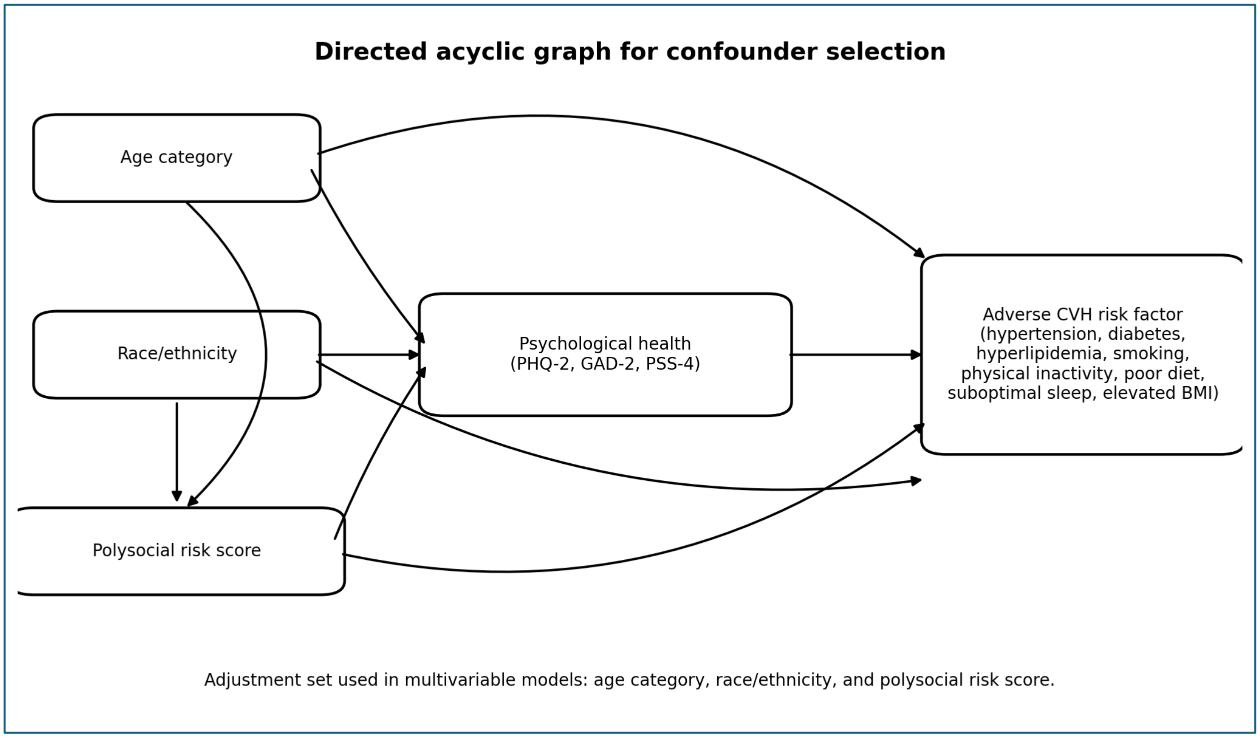


**Figure S1**. **Directed Acyclic Graph of the Hypothesized Relationships Among Psychological Health, Polysocial Risk, and Cardiovascular Health Risk Factors**
This directed acyclic graph illustrates the hypothesized relationships between psychological health and adverse cardiovascular health risk factors in women of reproductive age. Psychological health represents depressive symptoms, anxiety symptoms, and perceived stress, measured using the PHQ-2, GAD-2, and PSS-4, respectively. Adverse cardiovascular health risk factors represent hypertension, diabetes, hyperlipidemia, smoking, low physical activity, low fruit and vegetable intake, suboptimal sleep, and elevated body mass index. Based on this DAG, age category, self-reported race/ethnicity, and polysocial risk score were identified a priori as the minimally sufficient adjustment set for the multivariable models

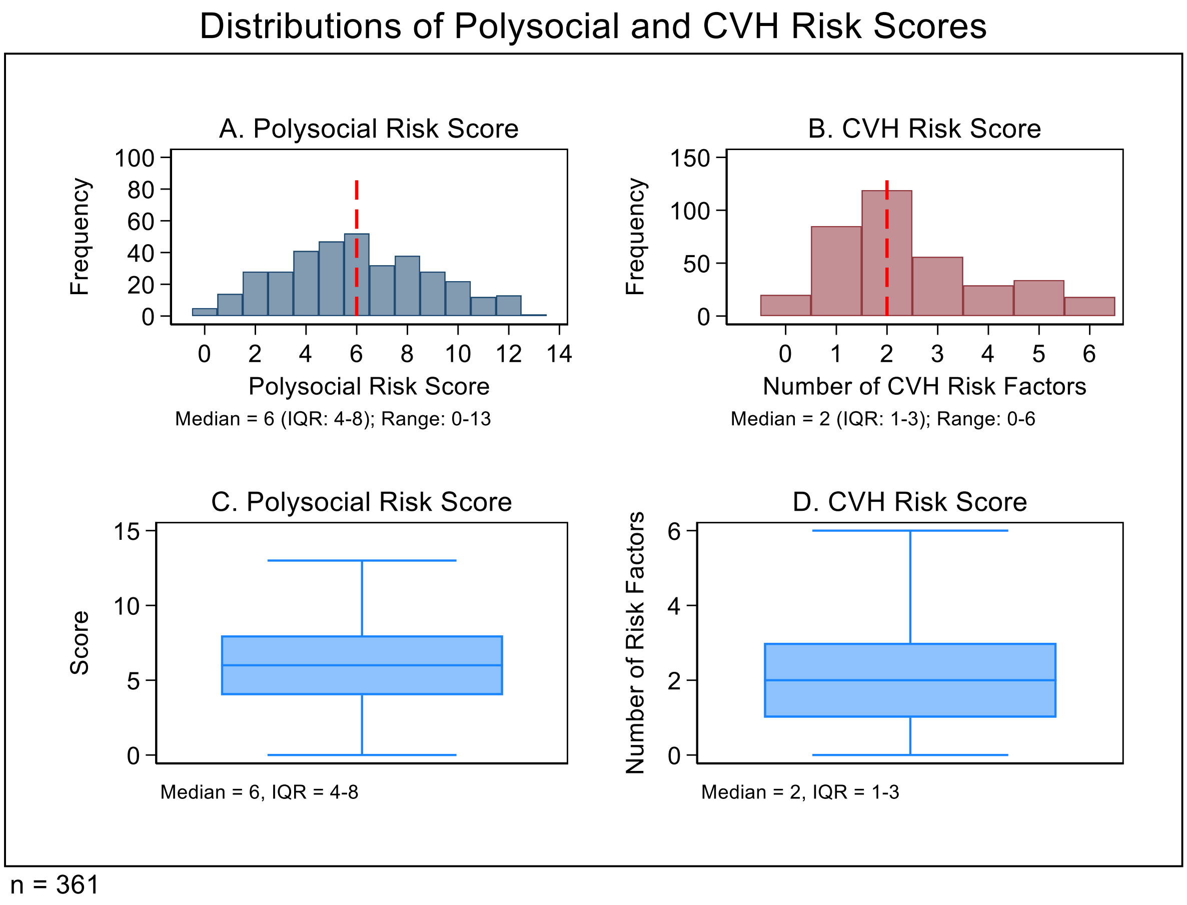


**Figure S2. Distributions of polysocial risk and cardiovascular health risk scores.** Histograms and boxplots showing the distribution of the polysocial risk score and the summed cardiovascular health (CVH) risk score in the analytic sample of women of reproductive age (n = 361). Panels A and C display the polysocial risk score, which ranged from 0 to 13, with a median of 6 and interquartile range (IQR) of 4 to 8. Panels B and D display the summed CVH risk score, which ranged from 0 to 6, with a median of 2 and IQR of 1 to 3. Dashed red lines in the histograms indicate the sample median.

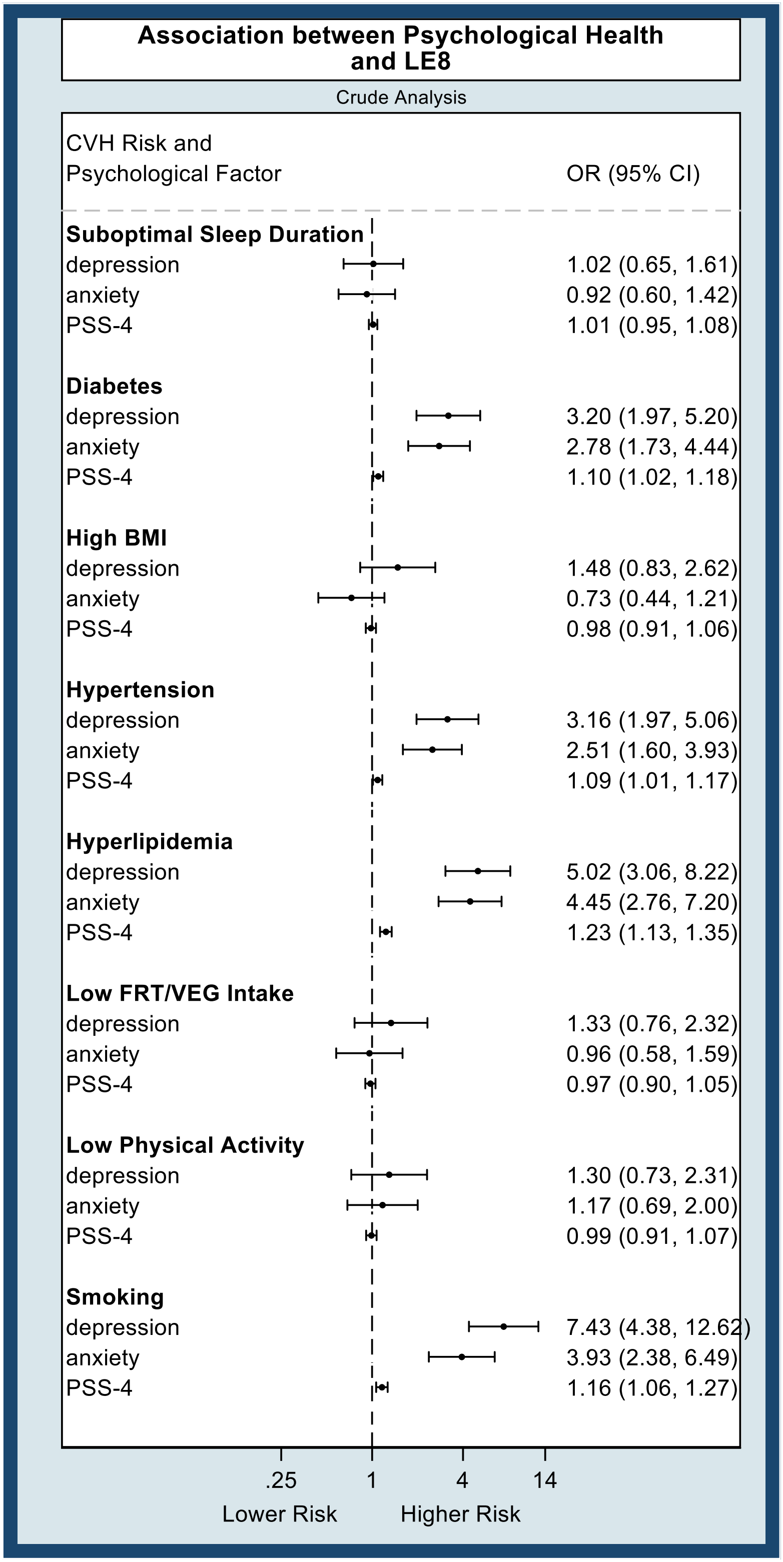
Association between Psychological Health and LE8
Crude Analysis
CVH Risk and Psychological Factor
OR (95% CI)
Suboptimal Sleep Duration
depression 1.02 (0.65, 1.61)
anxiety 0.92 (0.60, 1.42)
PSS-4 1.01 (0.95, 1.08)
Diabetes
depression 3.20 (1.97, 5.20)
anxiety 2.78 (1.73, 4.44)
PSS-4 1.10 (1.02, 1.18)
High BMI
depression 1.48 (0.83, 2.62)
anxiety 0.73 (0.44, 1.21)
PSS-4 0.98 (0.91, 1.06)
Hypertension
depression 3.16 (1.97, 5.06)
anxiety 2.51 (1.60, 3.93)
PSS-4 1.09 (1.01, 1.17)
Hyperlipidemia
depression 5.02 (3.06, 8.22)
anxiety 4.45 (2.76, 7.20)
PSS-4 1.23 (1.13, 1.35)
Low FRT/VEG Intake
depression 1.33 (0.76, 2.32)
anxiety 0.96 (0.58, 1.59)
PSS-4 0.97 (0.90, 1.05)
Low Physical Activity
depression 1.30 (0.73, 2.31)
anxiety 1.17 (0.69, 2.00)
PSS-4 0.99 (0.91, 1.07)
Smoking
depression 7.43 (4.38, 12.62)
anxiety 3.93 (2.38, 6.49)
PSS-4 1.16 (1.06, 1.27)
.25
1
4
14
Lower Risk
Higher Risk

**Figure S3. Association Between Psychological Health and Life's Essential 8 Cardiovascular Risk Factors (Unadjusted)**
The unadjusted associations between psychological health measures (high depressive symptoms, high anxiety symptoms, and high perceived stress) and adverse cardiovascular health risk factors as defined by Life's Essential 8. Cardiovascular risk factors were dichotomized as follows: hypertension, diabetes, and hyperlipidemia (physician-diagnosed); smoking (≥100 cigarettes in lifetime); low physical activity (<150 minutes/week); poor diet (<5 daily servings of fruits/vegetables); suboptimal sleep duration (<7 or >9 hours per night); and overweight/obesity (BMI ≥25 kg/m²).

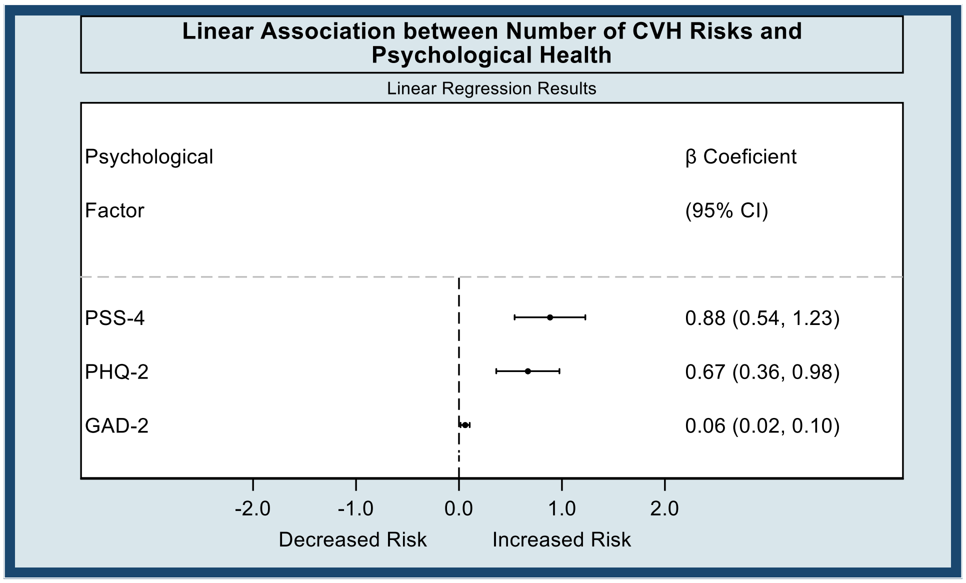


**Figure S4. Linear Association between Number of CVH Risk Factors and Psychological Health.** Forest plot showing the linear associations between continuous psychological health measures and the total number of adverse cardiovascular health (CVH) risk factors among women of reproductive age. Regression coefficients (β) and 95% confidence intervals are presented for perceived stress (PSS-4), depressive symptoms (PHQ-2), and anxiety symptoms (GAD-2). Higher PSS-4 and PHQ-2 scores were associated with a greater number of adverse CVH risk factors, with the largest coefficient observed for perceived stress. Higher GAD-2 scores were also positively associated with CVH risk burden, although the magnitude of association was smaller.

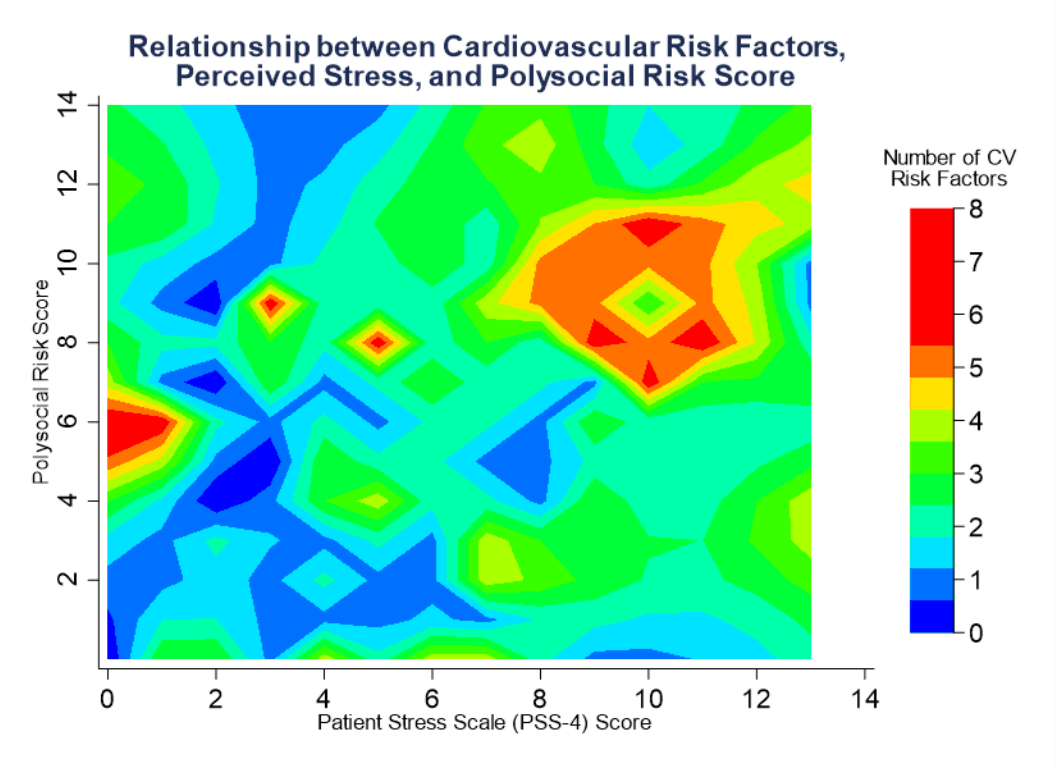


**Figure S5. Relationship Between Cardiovascular Risk Factors, Perceived Stress, and Polysocial Risk Scores**

Contour plot depicting the distribution of cardiovascular health risk factors by perceived stress (PSS-4 scores: 0–16) and polysocial risk scores (range: 0–14). Polysocial risk scores were calculated based on 14 social determinants of health across six domains: socioeconomic stability (education, employment, income, insurance, financial strain), living situation (housing stability, housing quality, marital status, homeownership), food security, transportation access, utilities, and interpersonal safety. Cardiovascular risk factors were assessed using Life's Essential 8, including hypertension, diabetes, hyperlipidemia, smoking, low physical activity (<150 min/week), suboptimal diet (<5 servings of fruits/vegetables per day), suboptimal sleep duration (<7 or >9 hours/night), and overweight/obesity (BMI $\geq$25 kg/m²).

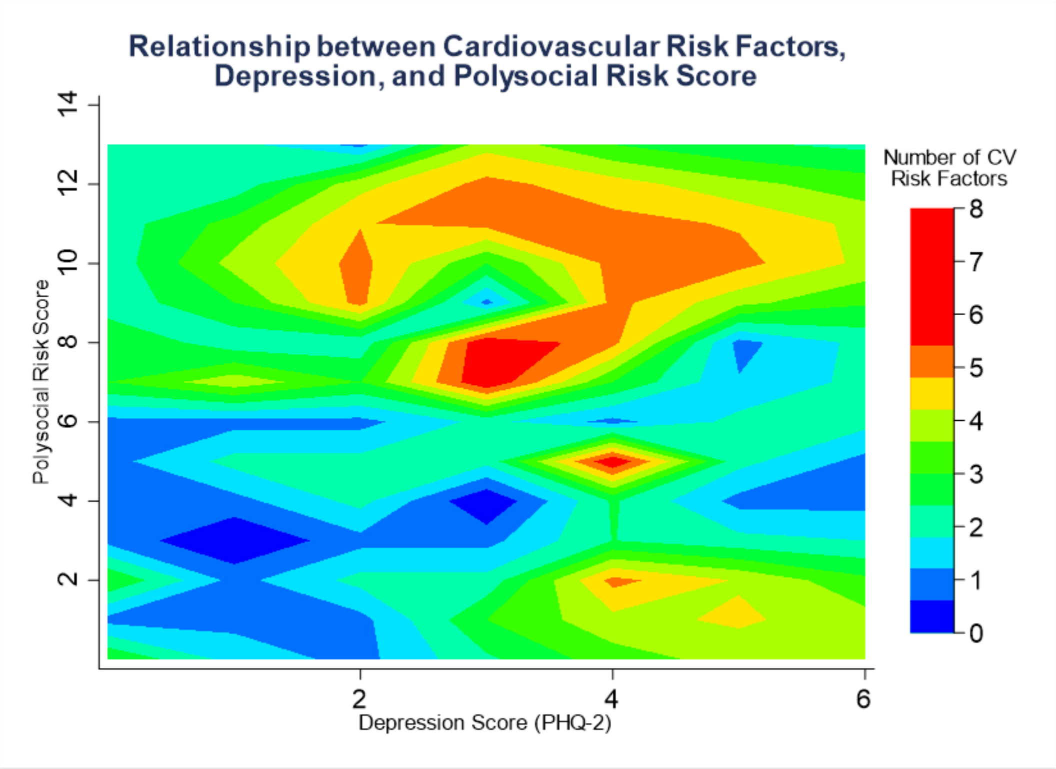


**Figure S6**. **Relationship Between Cardiovascular Risk Factors, Depression, and Polysocial Risk Scores**

Contour plot depicting the distribution of cardiovascular health risk factors by depression (PHQ-2 scores: 0–6) and polysocial risk scores (range: 0–14). Polysocial risk scores were calculated based on 14 social determinants of health across six domains: socioeconomic stability (education, employment, income, insurance, financial strain), living situation (housing stability, housing quality, marital status, homeownership), food security, transportation access, utilities, and interpersonal safety. Cardiovascular risk factors were assessed using Life's Essential 8, including hypertension, diabetes, hyperlipidemia, smoking, low physical activity (<150 min/week), suboptimal diet (<5 servings of fruits/vegetables per day), suboptimal sleep duration (<7 or >9 hours/night), and overweight/obesity (BMI ≥25 kg/m²).

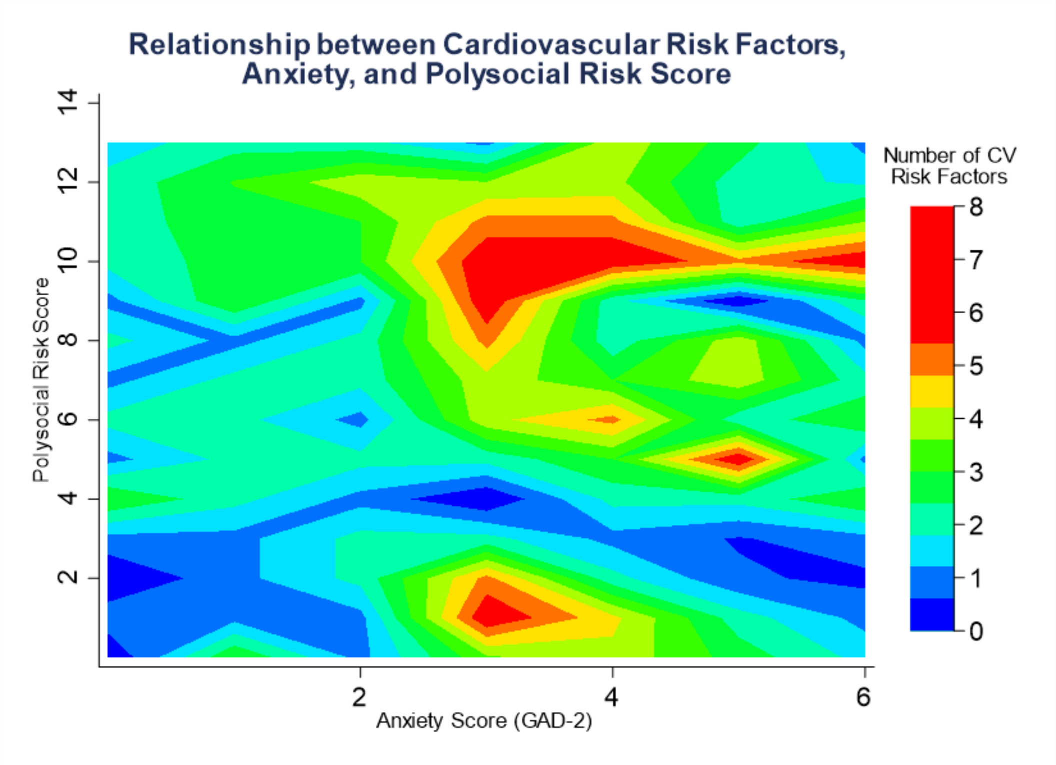


**Figure S7. Relationship Between Cardiovascular Risk Factors, Anxiety, and Polysocial Risk Scores.** Heat map depicting the distribution of cardiovascular health risk factors by anxiety (GAD-2 scores: 0–6) and polysocial risk scores (range: 0–14). Polysocial risk scores were calculated based on 14 social determinants of health across six domains: socioeconomic stability (education, employment, income, insurance, financial strain), living situation (housing stability, housing quality, marital status, homeownership), food security, transportation access, utilities, and interpersonal safety. Cardiovascular risk factors were assessed using Life's Essential 8, including hypertension, diabetes, hyperlipidemia, smoking, low physical activity (<150 min/week), suboptimal diet (<5 servings of fruits/vegetables per day), suboptimal sleep duration (<7 or >9 hours/night), and overweight/obesity (BMI ≥25 kg/m²).

**Table S1. Psychological Health Instruments**

| Component | PHQ-2 | GAD-2 | PSS-4 |
|---|---|---|---|
| **Construct assessed** | Depressive symptoms | Anxiety symptoms | Perceived stress |
| **Number of items** | 2 | 2 | 4 |
| **Recall period** | Past 2 weeks | Past 2 weeks | Past month |
| **Response options** | 0 = not at all; 1 = several days; 2 = more than half the | 0 = not at all; 1 = several days; 2 = more than half the | 0 = never; 1 = almost never; 2 = sometimes; |

| Component | PHQ-2 | GAD-2 | PSS-4 |
|---|---|---|---|
| | days; 3 = nearly every day | days; 3 = nearly every day | 3 = fairly often; 4 = very often |
| **Score range** | 0–6 | 0–6 | 0–16 |
| **Threshold/interpretation used in this study** | Score ≥3 indicated elevated depressive symptoms | Score ≥3 indicated elevated anxiety symptoms | Modeled continuously in regression analyses; dichotomized at the sample median (≥8 vs <8) for descriptive analyses only |
| **Item 1** | "Over the last 2 weeks, how often have you had little interest or pleasure in doing things?" | "Over the last 2 weeks, how often have you felt nervous, anxious, or on edge?" | "In the last month, how often have you felt that you were unable to control the important things in your life?" |
| **Item 2** | "Over the last 2 weeks, how often have you felt down, depressed, or hopeless?" | "Over the last 2 weeks, how often have you been unable to stop or control worrying?" | "In the last month, how often have you felt confident about your ability to handle your personal problems?"* |
| **Item 3** | — | — | "In the last month, how often have you felt that things were going your way?"* |
| **Item 4** | — | — | "In the last month, how often have you felt difficulties were piling up so high that you could not overcome them?" |
| **Notes** | Brief screening instrument derived from the PHQ-9; higher scores indicate greater depressive symptom burden | Brief screening instrument for generalized anxiety symptoms; higher scores indicate greater anxiety symptom burden | Not a diagnostic screening tool; higher scores indicate greater perceived stress |

**Table S2. Research Variables and Measurement**

| Research Variable | Measurement outcomes |
|---|---|
| **Dependent Variables: Cardiovascular Health as defined by Life's Essential 8** | |

| | |
|---|---|
| | Each factor will be scored as '1' for unfavorable outcomes and '0' for favorable outcomes.<br>Suboptimal CVH= ≥ 2 risk factors & optimal health= ≤ 1 CVD risk factors |
| Have you ever been told by a doctor that you had:<br>- Diabetes or high blood sugar (1)<br>- High cholesterol (1)<br>- High blood pressure (1) | 1: Yes<br>0: No |
| Have you smoked at least 100 cigarettes in your entire life? (1) | 1: Yes<br>0: No |
| Aside from your job, how many minutes of physical activity or exercise (such as running, walking, etc.) do you do per week? (1) | 1: Less than 150 minutes<br>0: 150 minutes or more |
| Not including fruit juices, how many servings of fruits/vegetables do you consume per day? (1) | 1: Less than 5<br>0: 5 or more |
| How many hours of sleep do you get per night? (1) | 1: More than 9 hours; Less than 7 hours<br>0: 7-9 hours |
| BMI: What is your weight & height? (1) | 1: Greater than 25<br>0: Less than or equal to 25 |
| **Independent Variables** | |
| **Individual SDoH** | |
| **Socioeconomic stability (5)** | |
| **Education**: What is the highest grade of formal or academic education have you had? | 1: High school graduate; Less than high school<br>0: Advanced degree; Some college; Some high school |
| **Employment:** We would like to know about what you do for work. For example, are you working full-time, working part-time, looking for work, retired, keeping house, or a student? | 1: Unemployed<br>0: Employed; Student |
| **Income:** What was the total combined income of your household in the past year? This can include income from wages, salaries, Social Security/retirement benefits, help from relatives, or other sources of income.<br><br>How many people are supported by this income right now? Count yourself and everyone supported by this income, even if they do not live in the same household as you. | Income was considered adverse if it fell below the following thresholds:<br><br>$15,060 for a household of 1<br>$20,440 for a household of 2<br>$25,820 for a household of 3<br>$31,200 for a household of 4<br>$36,580 for a household of 5<br>$41,960 for a household of 6<br>$47,340 for a household of 7<br>$52,720 for a household of 8<br><br>For households larger than 8, the threshold was calculated as $52,720 plus $5,380 for each additional household member. |
| **Financial strain:** How hard is it for you to pay for the very basics like food, housing, medical care, and heating? Would you say it is:* | 1: Very hard<br>0: Somewhat hard; Not hard at all |

| | |
|---|---|
| **Insurance:** Do you currently have health insurance? | 1: Uninsured<br>0: Government-sponsored or private insurance |
| **Living situation (4)** | |
| **Housing stability**: What is your living situation today?* | 1: Worried about losing housing; Unsteady place to live<br>0: Steady place to live |
| **Housing quality:** Think about the place you live. Do you have problems with any of the following? CHOOSE ALL THAT APPLY* | 1: Pests such as bugs, ants, or mice; Mold; Lead paint or pipes; Lack of heat; Oven or stove not working; Smoke detectors missing or not working; Water leaks; Other<br>0: None of the above |
| **Home ownership:** Please select the best way to describe the home where you live.* | 1:  Rented, Occupied without payment<br>0:  Owned |
| **Marital status:** What is your current marital status? | 1: Single; Divorced; Separated<br>0: Married; |
| **Food security (2):** | |
| Within the past 12 months, you worried that your food would run out before you got money to buy more.* | 1: Often true; Sometimes true<br>0: Never true |
| Within the past 12 months, the food you bought didn't last and you didn't have money to get more.* | 1: Often true; Sometimes true<br>0: Never true |
| **Transportation (1):** | |
| In the past 12 months, has lack of reliable transportation kept you from medical appointments, meetings, work or from getting things needed for daily living?* | 1: Yes<br>0: No |
| **Utilities (1):** | |
| In the past 12 months has the electric, gas, oil, or water company threatened to shut off services in your home?* | 1: Yes; Already shut off<br>0: No |
| **Interpersonal safety (1):** | |
| How often does anyone, including family and friends, physically hurt you?*<br><br>How often does anyone, including family and friends, insult or talk down to you?*<br><br>How often does anyone, including family and friends, threaten you with harm?*<br><br>How often does anyone, including family and friends, scream or curse at you?* | Continuous average of 4 questions<br><br>1: Scores greater than or equal to 11<br>0: Scores less than |
| **Cumulative SDoH** | |
| **Polysocial risk score:** A polysocial risk score will be calculated by summing the 14 individual SDoH. | The 14 identified SDoH will be assigned each a value of of '1' if unfavorable (e.g., uninsured), and '0' if favorable (e.g., insured).<br><br>Higher scores indicate more social risks. |
| *Accountable Health Communities Health-Related Social Needs Tool | |

**Table S3. Logistic Regression Diagnostic Summary**

| Outcome | Events | EPV | H-L $\chi^2$ | H-L p | BT-PSS p | BT-Poly p | Mean VIF |
|---|---|---|---|---|---|---|---|
| *Primary models (linear PSS-4, categorical age)* | | | | | | | |
| Low Physical Activity | 285 | 40.7 | 7.09 | 0.527 | 0.020* | 0.091 | 1.46 |
| Hypertension | 127 | 18.1 | 6.73 | 0.566 | 0.222 | 0.052 | 1.46 |
| Diabetes | 104 | 14.9 | 4.77 | 0.782 | 0.069 | 0.992 | 1.46 |
| Hypercholesterolemia | 106 | 15.1 | 14.17 | 0.077 | 0.263 | 0.391 | 1.46 |
| High BMI | 279 | 39.9 | 4.65 | 0.794 | 0.483 | 0.876 | 1.46 |
| Bad Sleep | 181 | 25.9 | 5.39 | 0.715 | 0.865 | 0.639 | 1.46 |
| Smoking | 88 | 12.6 | 6.10 | 0.636 | 0.321 | 0.158 | 1.46 |
| Low Fruit/Vegetable | 276 | 39.4 | 8.27 | 0.407 | 0.535 | 0.581 | 1.46 |
| *Spline-corrected sensitivity models* | | | | | | | |
| Low Physical Activity (linear spline, knot=9) | 285 | 35.6 | 5.58 | 0.695 | N/A | 0.121 | 1.44 |
| Diabetes (cubic spline, 3 knots) | 104 | 13.0 | 5.50 | 0.704 | N/A | 0.992 | 2.08 |

*Note. EPV = events per variable; H-L = Hosmer–Lemeshow goodness-of-fit test; BT = Box-Tidwell test for linearity in the logit; VIF = variance inflation factor. *p < 0.05. All models adjusted for depression, anxiety, PSS-4, age category, race, and polysocial risk score. BT-PSS is N/A in spline models, where PSS-4 non-linearity is addressed by the spline. The cubic spline mean VIF (2.08) reflects expected collinearity between basis functions; depression and anxiety VIFs remained low (1.58 and 1.47).*

**Table S4. Demographic and Clinical Characteristics by Age Group**

| | 18-25 | 26-34 | 35-50 | Total | P-value |
|---|---|---|---|---|---|
| | (N=80) (N=22.16) | (N=191) (N=52.91) | (N=90) (N=24.93) | (N=361) (N=100.00) | |
| (Age (Years) | 22.66 ± (1.80) (22.00-24.00) | 29.90 ± (2.54) (28.00-32.00) | 40.23 ± (3.57) (37.00-43.00) | 30.87 ± (6.68) (26.00-34.00) | <0.001 |
| minority_race | | | | | |
| 0 | 34 (42.50%) | 68 (35.60%) | 12 (13.33%) | 114 (31.58%) | <0.001 |
| 1 | 46 (57.50%) | 123 (64.40%) | 78 (86.67%) | 247 (68.42%) | |
| hisp_ethnicity | | | | | |
| 0 | 58 (72.50%) | 154 (80.63%) | 59 (65.56%) | 271 (75.07%) | 0.020 |
| 1 | 22 (27.50%) | 37 (19.37%) | 31 (34.44%) | 90 (24.93%) | |
| Race/Ethnicity | | | | | |
| Non-Hispanic White | 26 (32.50%) | 49 (25.65%) | 8 (8.89%) | 83 (22.99%) | <0.001 |
| Non-Hispanic Black | 27 (33.75%) | 99 (51.83%) | 44 (48.89%) | 170 (47.09%) | |

| | | | | | |
|---|---|---|---|---|---|
| Hispanics/Other* | 22 (27.50%) | 37 (19.37%) | 35 (38.89%) | 94 (26.04%) | |
| American Indian/Alaska Native | 0 (0.00%) | 1 (0.52%) | 1 (1.11%) | 2 (0.55%) | |
| Non-Hispanic Asian or Pacific Islander | 5 (6.25%) | 5 (2.62%) | 2 (2.22%) | 12 (3.32%) | |
| preglac12_bin | | | | | |
| No | 71 (88.75%) | 168 (87.96%) | 84 (93.33%) | 323 (89.47%) | 0.380 |
| Yes | 9 (11.25%) | 23 (12.04%) | 6 (6.67%) | 38 (10.53%) | |
| polysoc_marital | | | | | |
| 0 | 18 (22.50%) | 114 (59.69%) | 38 (42.22%) | 170 (47.09%) | <0.001 |
| 1 | 62 (77.50%) | 77 (40.31%) | 52 (57.78%) | 191 (52.91%) | |
| polysoc_edu | | | | | |
| 0 | 64 (80.00%) | 159 (83.25%) | 65 (72.22%) | 288 (79.78%) | 0.100 |
| 1 | 16 (20.00%) | 32 (16.75%) | 25 (27.78%) | 73 (20.22%) | |
| Income under $50,000 | | | | | |
| ≥$50,000 | 50 (62.50%) | 97 (50.79%) | 58 (64.44%) | 205 (56.79%) | 0.049 |
| <$50,000 | 30 (37.50%) | 94 (49.21%) | 32 (35.56%) | 156 (43.21%) | |
| polysoc_finstrain | | | | | |
| 0 | 35 (43.75%) | 59 (30.89%) | 45 (50.00%) | 139 (38.50%) | 0.005 |
| 1 | 45 (56.25%) | 132 (69.11%) | 45 (50.00%) | 222 (61.50%) | |
| polysoc_hinsur | | | | | |
| 0 | 69 (86.25%) | 147 (76.96%) | 71 (78.89%) | 287 (79.50%) | 0.222 |
| 1 | 11 (13.75%) | 44 (23.04%) | 19 (21.11%) | 74 (20.50%) | |
| polysoc_employ | | | | | |
| 0 | 71 (88.75%) | 156 (81.68%) | 87 (96.67%) | 314 (86.98%) | 0.002 |
| 1 | 9 (11.25%) | 35 (18.32%) | 3 (3.33%) | 47 (13.02%) | |
| polysoc_livsit | | | | | |
| Owned | 22 (27.50%) | 60 (31.41%) | 32 (35.56%) | 114 (31.58%) | 0.528 |
| Not owned | 58 (72.50%) | 131 (68.59%) | 58 (64.44%) | 247 (68.42%) | |
| polysoc_livsit1 | | | | | |
| 0 | 71 (88.75%) | 131 (68.59%) | 80 (88.89%) | 282 (78.12%) | <0.001 |
| 1 | 9 (11.25%) | 60 (31.41%) | 10 (11.11%) | 79 (21.88%) | |
| polysoc_livsit2 | | | | | |
| 0 | 35 (43.75%) | 66 (34.55%) | 26 (28.89%) | 127 (35.18%) | 0.124 |
| 1 | 45 (56.25%) | 125 (65.45%) | 64 (71.11%) | 234 (64.82%) | |
| polysoc_foodinsec1 | | | | | |
| 0 | 50 (62.50%) | 64 (33.51%) | 58 (64.44%) | 172 (47.65%) | <0.001 |
| 1 | 30 (37.50%) | 127 (66.49%) | 32 (35.56%) | 189 (52.35%) | |
| polysoc_foodinsec2 | | | | | |
| 0 | 53 (66.25%) | 69 (36.13%) | 56 (62.22%) | 178 (49.31%) | <0.001 |
| 1 | 27 (33.75%) | 122 (63.87%) | 34 (37.78%) | 183 (50.69%) | |
| polysoc_transport | | | | | |

| | | | | | |
|---|---|---|---|---|---|
| 0 | 56 (70.00%) | 108 (56.54%) | 80 (88.89%) | 244 (67.59%) | <0.001 |
| 1 | 24 (30.00%) | 83 (43.46%) | 10 (11.11%) | 117 (32.41%) | |
| polysoc_utilities | | | | | |
| 0 | 64 (80.00%) | 132 (69.11%) | 75 (83.33%) | 271 (75.07%) | 0.019 |
| 1 | 16 (20.00%) | 59 (30.89%) | 15 (16.67%) | 90 (24.93%) | |
| polysoc_safety | | | | | |
| 0 | 66 (82.50%) | 136 (71.20%) | 87 (96.67%) | 289 (80.06%) | <0.001 |
| 1 | 14 (17.50%) | 55 (28.80%) | 3 (3.33%) | 72 (19.94%) | |
| cvh_diabetes | | | | | |
| 0 | 70 (87.50%) | 118 (61.78%) | 69 (76.67%) | 257 (71.19%) | <0.001 |
| 1 | 10 (12.50%) | 73 (38.22%) | 21 (23.33%) | 104 (28.81%) | |
| cvh_hichol | | | | | |
| 0 | 65 (81.25%) | 120 (62.83%) | 70 (77.78%) | 255 (70.64%) | 0.002 |
| 1 | 15 (18.75%) | 71 (37.17%) | 20 (22.22%) | 106 (29.36%) | |
| cvh_hibp | | | | | |
| 0 | 60 (75.00%) | 114 (59.69%) | 60 (66.67%) | 234 (64.82%) | 0.050 |
| 1 | 20 (25.00%) | 77 (40.31%) | 30 (33.33%) | 127 (35.18%) | |
| cvh_smoking | | | | | |
| 0 | 76 (95.00%) | 120 (62.83%) | 77 (85.56%) | 273 (75.62%) | <0.001 |
| 1 | 4 (5.00%) | 71 (37.17%) | 13 (14.44%) | 88 (24.38%) | |
| cvh_lowphys | | | | | |
| 0 | 17 (21.25%) | 38 (19.90%) | 21 (23.33%) | 76 (21.05%) | 0.804 |
| 1 | 63 (78.75%) | 153 (80.10%) | 69 (76.67%) | 285 (78.95%) | |
| cvh_lowfrtveg | | | | | |
| 0 | 22 (27.50%) | 39 (20.42%) | 24 (26.67%) | 85 (23.55%) | 0.330 |
| 1 | 58 (72.50%) | 152 (79.58%) | 66 (73.33%) | 276 (76.45%) | |
| cvh_badsleep | | | | | |
| 0 | 41 (51.25%) | 105 (54.97%) | 34 (37.78%) | 180 (49.86%) | 0.026 |
| 1 | 39 (48.75%) | 86 (45.03%) | 56 (62.22%) | 181 (50.14%) | |
| cvh_hibmi | | | | | |
| 0 | 32 (40.00%) | 40 (20.94%) | 10 (11.11%) | 82 (22.71%) | <0.001 |
| 1 | 48 (60.00%) | 151 (79.06%) | 80 (88.89%) | 279 (77.29%) | |
| Dichotomized PHQ-2 Score (>=3 high) | | | | | |
| low | 67 (83.75%) | 108 (56.54%) | 81 (90.00%) | 256 (70.91%) | <0.001 |
| high | 13 (16.25%) | 83 (43.46%) | 9 (10.00%) | 105 (29.09%) | |
| Dichotomized GAD-2 Score (>=3 high) | | | | | |
| low | 48 (60.00%) | 110 (57.59%) | 74 (82.22%) | 232 (64.27%) | <0.001 |
| high | 32 (40.00%) | 81 (42.41%) | 16 (17.78%) | 129 (35.73%) | |
| Dichotomized PSS-4 Score (>=8 high) | | | | | |

| | | | | | |
|---|---|---|---|---|---|
| low | 30 (37.50%) | 67 (35.08%) | 35 (38.89%) | 132 (36.57%) | 0.810 |
| high | 50 (62.50%) | 124 (64.92%) | 55 (61.11%) | 229 (63.43%) | |
| PHQ-2 | 1.54 ± (1.34) (0.00-2.00) | 2.15 ± (1.58) (1.00-3.00) | 0.88 ± (1.31) (0.00-2.00) | 1.70 ± (1.55) (0.00-3.00) | <0.001 |
| GAD-2 | 2.12 ± (1.88) (0.00-3.00) | 2.21 ± (1.58) (1.00-3.00) | 1.26 ± (1.60) (0.00-2.00) | 1.96 ± (1.70) (0.00-3.00) | <0.001 |
| PSS-4 | 6.71 ± (3.28) (5.00-9.00) | 6.79 ± (3.05) (6.00-9.00) | 6.23 ± (3.49) (4.00-9.00) | 6.63 ± (3.21) (5.00-9.00) | 0.532 |
| safety_score | 6.71 ± (3.51) (4.00-8.00) | 8.40 ± (4.21) (4.00-11.00) | 5.19 ± (2.39) (4.00-5.00) | 7.22 ± (3.91) (4.00-10.00) | <0.001 |
| Polysocial Risk Score | 5.60 ± (2.74) (4.00-7.50) | 6.51 ± (2.86) (5.00-9.00) | 5.28 ± (2.98) (3.00-7.00) | 6.00 ± (2.91) (4.00-8.00) | <0.001 |
| cvhrisk_number | 1.70 ± (1.21) (1.00-2.00) | 2.77 ± (1.69) (1.00-4.00) | 2.44 ± (1.22) (2.00-3.00) | 2.45 ± (1.54) (1.00-3.00) | <0.001 |
| ideal_cvh | | | | | |
| 0 | 44 (55.00%) | 142 (74.35%) | 70 (77.78%) | 256 (70.91%) | 0.002 |
| 1 | 36 (45.00%) | 49 (25.65%) | 20 (22.22%) | 105 (29.09%) | |
| Mean (Standard deviation) or Median (Interquartile Range) shown for continuous variables: p-value from Kruskal-Wallis rank test. Frequency (Percent%) shown for categorical variables: p-value from Chi-square tests. | | | | | |

**Table S5. Cross-classification by depression, anxiety, and/or stress (N = 361)**

| Any Psychological Distress | No CVH risk factors | ≥1 CVH risk factor | Total |
|---|---|---|---|
| **No distress** | 7 (1.9%) | 112 (31.0%) | 119 (33.0%) |
| **Any distress** | 13 (3.6%) | 229 (63.4%) | 242 (67.0%) |
| **Total** | **20 (5.5%)** | **341 (94.5%)** | **361 (100%)** |

*Note. Distress defined as screening positive for depression (PHQ-2 ≥ 3), anxiety (GAD-2 ≥ 3), and/or stress (PSS-4 ≥ 8, the sample median). PSS-4 dichotomization was applied for this descriptive cross-classification only; it remained continuous in all regression analyses.*

**Table S6. Adjusted Logistic Regression Models for Individual Cardiovascular Health Risk Factors**

| **Predictor** | **Low PA** | **Hyper-tension** | **Diabetes** | **Hyper-cholesterolemia** | **High BMI** | **Bad Sleep** | **Smoking** | **Low FRT/VEG** |
|---|---|---|---|---|---|---|---|---|
| *aOR (95% CI)* | *aOR (95% CI)* | *aOR (95% CI)* | *aOR (95% CI)* | *aOR (95% CI)* | *aOR (95% CI)* | *aOR (95% CI)* | *aOR (95% CI)* | *aOR (95% CI)* |
| ***Psychological Health*** | | | | | | | | |
| Depression (PHQ-2) | | | | | | | | |
| *Low (< 3)* | *1.00 (Reference)* | *1.00 (Reference)* | *1.00 (Reference)* | *1.00 (Reference)* | *1.00 (Reference)* | *1.00 (Reference)* | *1.00 (Reference)* | *1.00 (Reference)* |
| High (≥ 3) | 1.25 (0.61, 2.57) | 1.64 (0.89, 3.04) | 1.29 (0.67, 2.48) | 2.00* (1.06, 3.76) | 1.46 (0.71, 3.00) | 1.13 (0.63, 2.03) | 3.45*** (1.71, 6.95) | 1.25 (0.62, 2.53) |
| Anxiety (GAD-2) | | | | | | | | |

| | | | | | | | | |
|---|---|---|---|---|---|---|---|---|
| *Low (< 3)* | *1.00 (Reference)* | *1.00 (Reference)* | *1.00 (Reference)* | *1.00 (Reference)* | *1.00 (Reference)* | *1.00 (Reference)* | *1.00 (Reference)* | *1.00 (Reference)* |
| High (≥ 3) | 1.07 (0.56, 2.03) | 1.68 (0.94, 2.99) | 1.88* (1.02, 3.49) | 2.52** (1.39, 4.56) | 0.61 (0.32, 1.15) | 0.93 (0.55, 1.58) | 1.88 (0.95, 3.72) | 0.80 (0.43, 1.48) |
| PSS-4 (per 1-point increase) | 0.98 (0.89, 1.06) | 1.00 (0.92, 1.09) | 1.00 (0.92, 1.10) | 1.13* (1.02, 1.25) | 0.98 (0.90, 1.07) | 1.02 (0.95, 1.09) | 1.03 (0.92, 1.14) | 0.97 (0.90, 1.06) |
| ***Demographics*** | | | | | | | | |
| Age group (years) | | | | | | | | |
| *18–25* | *1.00 (Reference)* | *1.00 (Reference)* | *1.00 (Reference)* | *1.00 (Reference)* | *1.00 (Reference)* | *1.00 (Reference)* | *1.00 (Reference)* | *1.00 (Reference)* |
| 26–34 | 1.03 (0.52, 2.01) | 1.38 (0.72, 2.65) | 3.35** (1.54, 7.30) | 1.95 (0.95, 4.01) | 2.10* (1.14, 3.84) | 0.79 (0.46, 1.39) | 7.92*** (2.62, 23.95) | 1.46 (0.76, 2.78) |
| 35–50 | 0.91 (0.43, 1.94) | 1.41 (0.67, 2.95) | 2.18 (0.90, 5.32) | 1.56 (0.67, 3.64) | 5.10*** (2.23, 11.69) | 1.58 (0.83, 3.00) | 3.36 (0.96, 11.73) | 1.12 (0.55, 2.29) |
| Race/ethnicity | | | | | | | | |
| *Non-Hispanic White* | *1.00 (Reference)* | *1.00 (Reference)* | *1.00 (Reference)* | *1.00 (Reference)* | *1.00 (Reference)* | *1.00 (Reference)* | *1.00 (Reference)* | *1.00 (Reference)* |
| Non-Hispanic Black | 0.94 (0.48, 1.82) | 2.43** (1.27, 4.64) | 3.12** (1.52, 6.40) | 1.73 (0.87, 3.42) | 0.91 (0.47, 1.73) | 1.25 (0.72, 2.17) | 3.98** (1.74, 9.12) | 0.61 (0.32, 1.17) |
| Hispanic/Other | 1.00 (0.46, 2.15) | 2.27* (1.09, 4.71) | 1.72 (0.75, 3.96) | 2.00 (0.92, 4.36) | 1.00 (0.47, 2.12) | 1.92* (1.02, 3.61) | 2.37 (0.90, 6.19) | 0.95 (0.44, 2.06) |
| American Indian/Alaska Native | — | — | — | 1.56 (0.08, 31.36) | — | 1.07 (0.06, 18.56) | — | — |
| Non-Hispanic Asian/Pacific Islander | 1.40 (0.28, 7.06) | — | 2.13 (0.40, 11.41) | — | 0.37 (0.10, 1.35) | 0.74 (0.20, 2.71) | 2.23 (0.24, 20.78) | 3.33 (0.40, 27.92) |
| ***Social Determinants*** | | | | | | | | |
| Polysocial risk score (per 1-point) | 1.01 (0.92, 1.12) | 1.16** (1.06, 1.27) | 1.16** (1.05, 1.28) | 1.08 (0.98, 1.19) | 1.11* (1.00, 1.24) | 1.05 (0.97, 1.14) | 1.07 (0.96, 1.20) | 1.08 (0.98, 1.19) |
| ***Model fit*** | | | | | | | | |
| N | 359 | 347 | 359 | 349 | 359 | 361 | 359 | 359 |
| Pseudo $R^2$ | 0.005 | 0.106 | 0.147 | 0.167 | 0.081 | 0.033 | 0.261 | 0.031 |
| Log likelihood | -184.5 | -202.8 | -182.7 | -178.6 | -177.2 | -242.0 | -146.0 | -190.4 |
| LR $\chi^2$ | 1.67 | 48.03 | 63.05 | 71.46 | 31.33 | 16.40 | 103.34 | 12.20 |

*Note. Values are adjusted odds ratios (aOR) with 95% confidence intervals from logistic regression models. Each model included depression, anxiety, PSS-4, age category, race/ethnicity, and the polysocial risk score as predictors. Reference categories (in italics) were assigned an OR of 1.00. Em dashes (—) indicate that the category was not estimable in that model due to small cell size or perfect prediction. Significance: *p < 0.05, **p < 0.01, ***p < 0.001. PHQ-2 = Patient Health Questionnaire-2; GAD-2 = Generalized Anxiety Disorder-2; PSS-4 = Perceived Stress Scale (4-item); FRT/VEG = fruit/vegetable; BMI = body mass index; PA = physical activit*
